# Sodium Citrate as a Preventive of Milk Coagulation

**Published:** 1914-08

**Authors:** 


**Sodium Citrate as a Preventive of Milk Coagulation.** A. W. Bosworth and Lucius L. Van Slyke, Am. Jour. of Dis. of Children, April, 1914, explain that in place of calcium casenate of normal milk, there is a more soluble, double salt of calcium and sodium. In the presence of rennin, a paracasenare is formed which is insoluble for the normal milk, soluble if sodium citrate has been added. 4 milligrams of sodium citrate per 100 c.c. of milk is sufficient to prevent dense curds. (Note: In digestion experiments the gastric filtrate sometimes fails to coagulate milk even when peptonization of egg albumin occurs. This has been taken as an indication of a specific failure of rennin. There is, however, a minority opinion that pepsin and rennin are identical. At any rate, specific failure of one test without failure of the other is quite rare and reliance should not be placed on a negative result of the milk coagulation test until it has been controlled with milk known to be coagulable with normal gastric filtrate. Sodium bicarbonate is sometimes added to milk to check fermentation and it is quite possible that diet or impregnation of water with sodium salts may produce a milk containing the double casenate of calcium and sodium.

